# Supplementary figures and images for: A systematic approach to the scale separation problem in the development of multiscale models
Source: PLoS One. 2021 May 18;16(5):e0251297. doi: 10.1371/journal.pone.0251297 (PMC8130972; doi:10.1371/journal.pone.0251297)

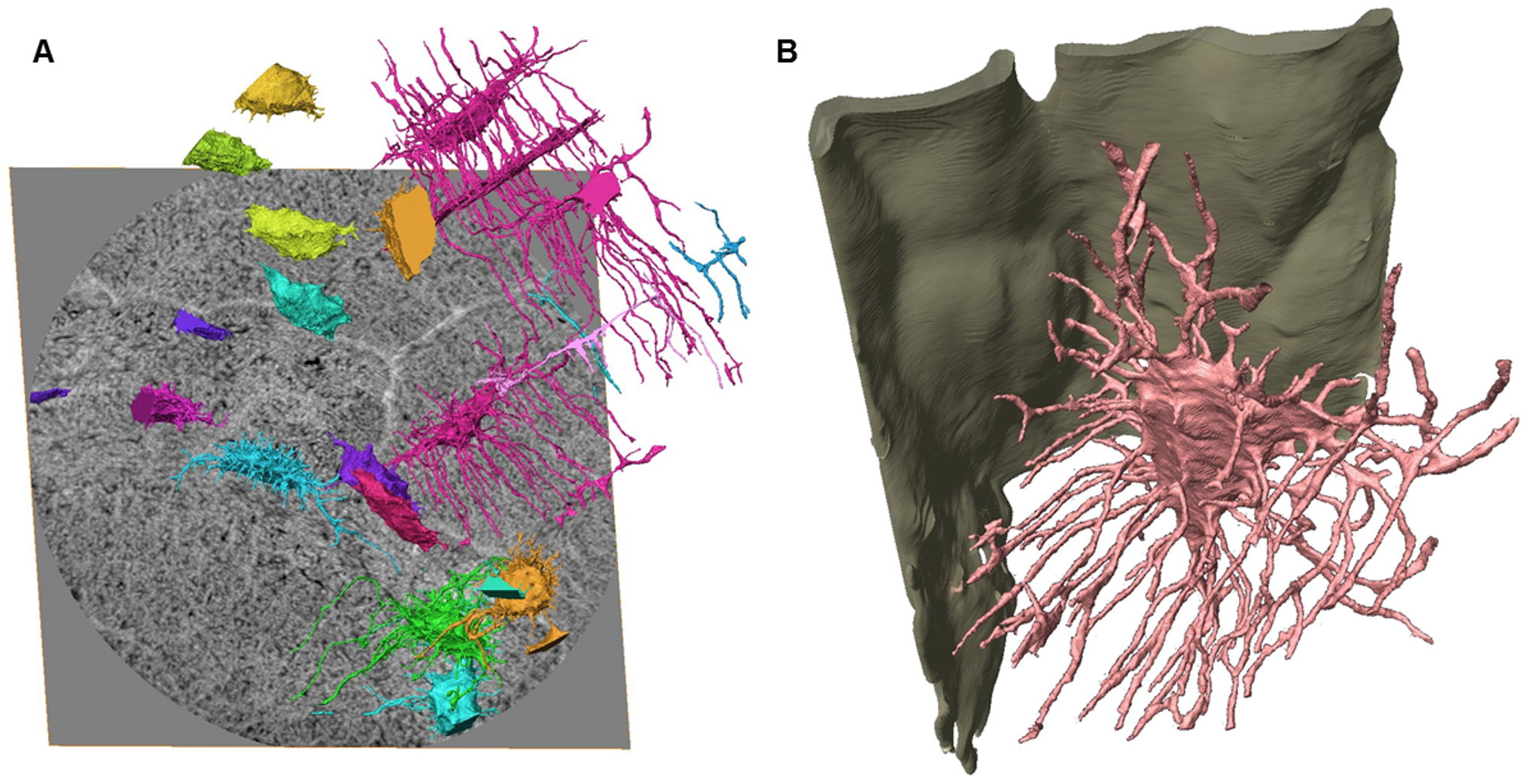

Supplement: S1A Fig — (TIF) [file pone.0251297.s001.tif]

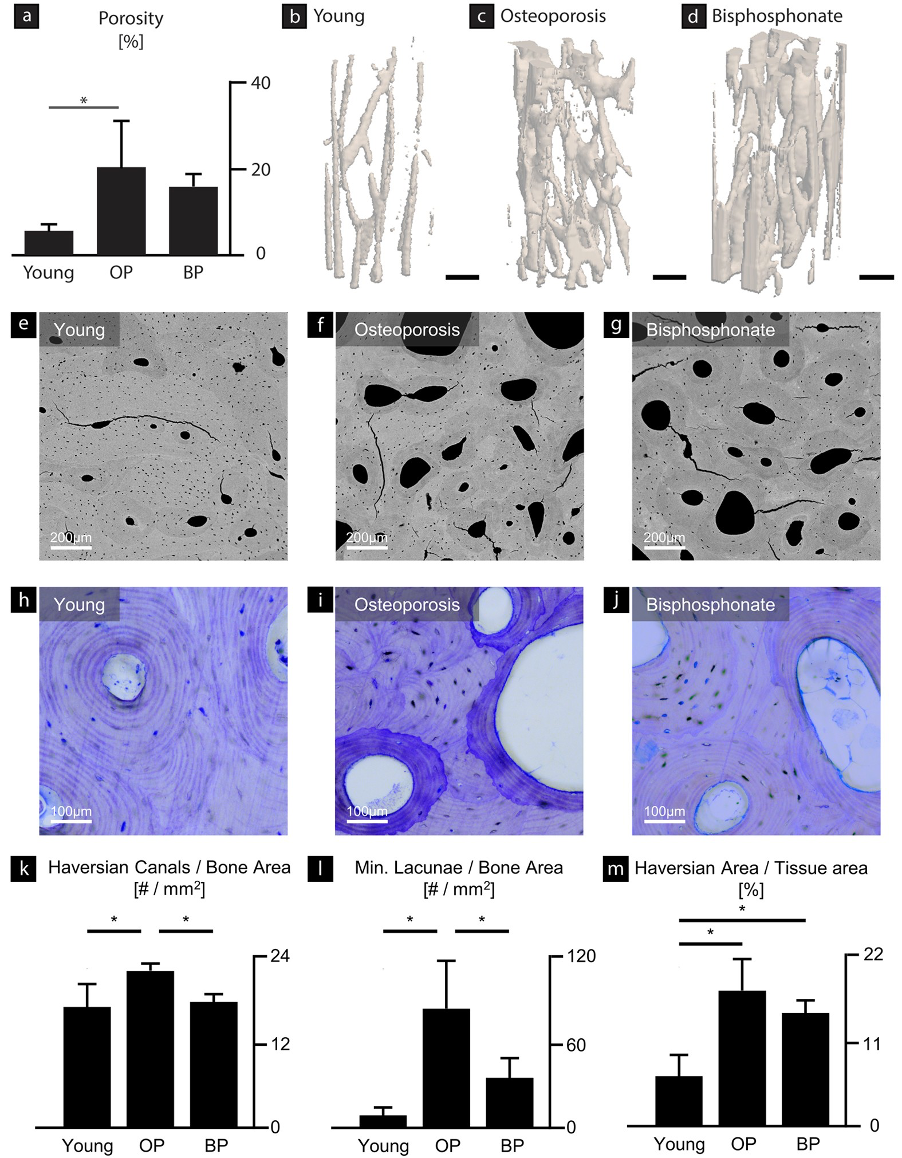

Supplement: S1B Fig — (PNG) [file pone.0251297.s002.png]
