# Supplementary material for: A systematic approach to the scale separation problem in the development of multiscale models
Source: PLoS One. 2021 May 18;16(5):e0251297. doi: 10.1371/journal.pone.0251297 (PMC8130972; doi:10.1371/journal.pone.0251297)
Supplement: S1 File — (DOCX) [file pone.0251297.s003.docx]

Supporting Information

# Overview

In this section multiscale model formulations are presented where the portions of reality considered for inclusion in the model are representative of the state-of-the-art. Both model formulations correspond to prediction of future femur strength. Section S2 considers the problem in a living human and strength in a fall orientation, while section S3 considers the problem in a living mouse and strength under three-point bending [1]. Computability of the models is considered outside of the scope of the present study. Note that symbols introduced in the following two sections, even when identical in appearance to those in the main text, possess the definitions as given below and not as in the main text.

# Femur strength after 10 years in a living human

## Description of the closed system

Consider a femur B, occupying a connected region *Ω* in **R**^3^, bounded by the surface *Γ* and possessing a maximum size *L** ~ 10^0^ m (typical length of an adult human femur). The femur is contained fully and at all times within an organism with which it interacts both mechanically and biochemically. Mechanical energy of the organism increases either internally (conversion from biochemical energy through muscles contractility) or externally (due to organism–environment interaction). Irrespective of where the mechanical energy is produced, it finally results in a distribution of tractions on the boundary of the bone always via an interaction between the bone and the organism. It is assumed that the forces on the boundary *Γ* are a known spatiotemporally varying field. It is assumed that the transport of biochemical material and energy from the organism to the bone (and vice versa) are known as spatiotemporally varying biochemical signals throughout *Ω*, and that the organism can sustain the metabolic rate needed to produce these signals.

The bone region is assumed to comprise two material phases: a bone matrix phase and a non-bone matrix phase. Bone matrix comprises hydroxyapatite foam embedded with mineralized wet collagen fibrils and is the primary load-bearing constituent of bone. The non-bone matrix phase comprises vascular material found in the Haversian system and bone marrow and extravascular material occupying pore spaces such as osteocytic lacunae [2]. There is some debate in literature on the mechanical response of the non-bone matrix phase. Fritsch and Hellmich [2] assumed that the extravascular material in lacunar pore spaces have water as the main constituent and that these spaces are not inter-connected. Thereby, some contribution to resistance to volumetric strains may be expected through the bulk modulus of water. In contrast, more recent studies [3-5] mostly agree to consider lacunar and canalicular porosities to be connected such that the fluid permeates as in a porous medium following Darcy’s law and can provide some limited hydraulic stiffening. Neither consideration fundamentally limits the following analysis; as both can be reconciled by substituting the purely elastic description for the bone matrix by a porous medium description (with entrapped and/or free fluid) of the bone composite (matrix/non-matrix). Thus, for the sake of simplicity, it is assumed here that only the bone matrix­ phase contributes to the mechanical stiffness of the bone.

Let the regions occupied by the bone matrix, the non-bone matrix phases be denoted by *Ω*_bm_, *Ω*_lac_ and *Ω*_vas_ respectively and occupying mutually exclusive and exhaustive sub-volumes of *Ω*. It is assumed that *Ω*_lac_ and *Ω*_vas_ are connected, hence we denote *Ω*_bm′_ = *Ω*_lac_ ∪ *Ω*_vas_. The boundary shared by *Ω* and *Ω*_bm_ is denoted by *Γ*_bm_; boundary *Γ*_bm′_ is similarly defined (Fig S1). The internal boundary between *Ω*_bm_ and *Ω*_bm′_ is denoted by *Γ*_bm–bm′_ and is assumed to be a connected manifold. It is assumed that features that vary over characteristic distances smaller than *l** = 0.1 μm = 10^–7^ m do not influence the mechanical or biochemical response of the bone. Notice that this assumption does not fail to account for the existence of microcracks. The surface of a microcrack is essentially part of the surface *Γ*_bm–bm′_ defined above; we only assume that microcrack features that vary over distances smaller than *l** do not influence bone strength. The above restriction to minimum distances of length *l** includes the following features of adult human bones: osteons (193±11 μm in diameter [7]), Haversian and Volkmann canals (77±21 μm in diameter [8]), inter-trabecular porosities (~ 500 μm in diameter) and lacunae (70±36 μm in diameter [9]) but excludes canaliculi (150–550 nm in diameter [10]). The materials composing region *Ω* are assumed to be continua down to *l**. This implies that for any two points that are separated by a distance smaller than *l**, variables of interest taken at these points (e.g. location, stress, traction, displacement and strain) cannot be distinguished from each other. Impact forces applied on the femur surface (such as during a fall to the side) and strains within the femur possess characteristic temporal variations in the range of 1–1000 ms [11, 12]. The aim of the model is to determine the strength of the femur after 10 years (=10^9^ s) from present. Thus, the domain of observation ranges between 10^–7^ m to ~10^0^ m in length and from 10^–3^ s to ~10^9^ s in time.

Fig S1. Features of bone observable using instruments with different spatial resolutions

(A) Cortical bone region from the femoral midshaft of an elderly woman [13]. A slice of X-ray phase nanotomography image (0.06 μm resolution, grayscale indicating mass density) is shown, overlayed with three-dimensional rendering of lacunar porosities (belonging to *Ω*_lac_) and canaliculi (included in *Ω*_bm_) in the whole imaged volume (maximum length 90 µm). Distinct colours indicate unconnected components. (B) Cortical bone region from the femoral midshaft of an elderly woman [14]. The bone region shown corresponds to *Ω*_[3]m_ and comprises sub-volumes *Ω*_[2]m_ (bright regions on the left, appearing in the two-dimensional backscattered scanning electron microscopy image) and *Ω*_[2]v_ (coloured three-dimensional region on the right, reconstructed from micro-CT image with 10 μm resolution). (C) A frontal cross-section of the proximal femur of an elderly woman based on clinical quantitative CT images taken at 0.625 mm resolution, as described in [15]. Regions containing only the vascular phase (*Ω*_[3]v_, volumetric bone mineral density < 0.1 g/cm^3^) are removed. The region shown (*Ω*_[3]m_) comprises bone matrix, extravascular and vascular phases. The transition from blue to red corresponds to increase in the local bone matrix phase content.

## Abstraction of the closed system

It is assumed that the bone matrix phase is biochemically inert, hence all biochemical activity that gives rise to bone remodelling occurs only in other phases. At the same time, the mechanical stiffness of region *Ω* is assumed to arise only from the mechanical stiffness of the bone matrix phase, while the other phases are assumed to possess no ability to store mechanical energy. In other words, the boundary *Γ*_bm–bm′_ partitions *Ω* into two distinct regions *Ω*_bm_ and *Ω*_bm′_: the first mechanically responsive but biochemically inert; the second mechanically inert but biochemically active. While this assumption contradicts what we know of bone biology, in that bone cells exist entrapped inside the extra-cellular matrix, yet while being chemically active these cells do not appose or resorb bone matrix. Within the limits of these considerations, such assumption is thereby acceptable.

Consider an infinitesimally small region d*Ω* contained in *Ω* and bounded by the surface d*Γ*, and all biochemical activity to be absent everywhere within d*Ω*. The shape of the sub-region d*Ω*_bm_ changes over time in response to known time-varying surface tractions and displacements applied on d*Γ*_bm_, while the boundary d*Γ*_bm′_ remains traction-free. It is assumed that the contribution from inertial stresses can be neglected [16]. Mechanical equilibrium in d*Ω*_bm_ is mathematically stated as [17, 18]

| Div *σ* = 0 | (1) |
| --- | --- |

Here, Div is the reference configuration divergence operator and *σ* is the second Piola–Kirchhoff (PK) stress tensor.

The bone matrix phase is treated as a heterogeneous, isotropic material under large strain. The material is assumed to be initially linear elastic followed by non-hardening plastic behaviour and progressive damage (both plasticity and damage are assumed history-independent) [19]. The isotropy assumption is undermined by experimental observations [20] which highlight the role of the preferential orientation of bone mineral crystals within the bone matrix [21]. Yet, this limitation is retained here for the simplicity of mathematical description and is easily removed by extending the model using well-established theories [22]. The assumption of elastoplastic damage is consistent with experimental observations of strain-controlled fracture of the bone matrix [23, 24], and can be extended to include history-dependence and hardening [19]. Spatial heterogeneity allows for distinguishing the elastic and/or post-elastic responses of bone matrix in regions such as cement lines, interstitial lamellae and osteonal and trabecular lamellae [20, 25]. Given the above assumptions, the stress and strain rates are related by the constitutive law [26]

| $\dot{\text{σ}}\text{ }\text{=}\text{ }\text{H}^{\text{epd}}\text{:}\dot{\text{E}}$ | (2) |
| --- | --- |

where, the superscripted dot ˙ refers to a differentiation with respect to time, *H*^epd^ is the elastoplastic-damage modulus tensor, the operator : refers to a double contraction tensor product, *E* = ½ (*F*^T^*F* – *I*) is the Green–Lagrange (GL) strain tensor, *F* = *I* + Grad *u* is the deformation gradient tensor, the superscript T indicates a transpose, *I* is the second-order unit tensor, Grad is the gradient operator in the reference configuration and *u* is the displacement vector. The modulus tensor *H*^epd^ depends on the current stress *σ* and strain *E* and is parameterised by the undamaged elastic modulus tensor *H*^(0)^, the yield stress *K* and a damage evolution function *d*.

One can obtain *σ* and *u* everywhere in d*Ω*_bm_ as a solution of equations (1) and (2) supplemented by boundary conditions:

| *F*∙*σ*∙*N* = *τ* | on the traction boundary d*Γ*_bm,_*_τ_* | (3a) |
| --- | --- | --- |
| *u* = *a* | on the displacement boundary d*Γ*_bm,_*_u_* | (3b) |
| *F*∙*σ*∙*N* = 0 | on the internal boundary d*Γ*_bm–bm′_ | (3c) |

where, *N* and *τ* are respectively the (outward) normal and the traction vectors at any point of the surface d*Γ*_bm,_*_τ_* (in the reference configuration), *a* is the displacement applied at any point of the surface d*Γ*_bm,_*_u_* and d*Γ*_bm,_*_τ_* and d*Γ*_bm,_*_u_* are mutually exclusive and exhaustive sub-surfaces of d*Γ*_bm_. Notice that the displacement of the internal boundary d*Γ*_bm–bm′_ is obtained as part of the solution.

Spatial heterogeneity of the constitutive law parameters (*H*^(0)^, *K* and *d*) follow the local material composition of the bone matrix. Here, we only explicitly state the dependence of the undamaged elastic modulus tensor on bone matrix density

| *H*^(0)^ = *H*^(0)^ (*ρ*) | (4) |
| --- | --- |

Biochemical activity changes the phase composition in the region *Ω*. It is assumed that phase transformations occur only in the neighbourhood of the existing phase-partition boundary *Γ*_bm–bm′_, such that the new boundary remains connected. In particular, this assumption avoids the possibility of creating new bone matrix regions fully encapsulated within non-bone matrix regions, or vice versa. Only the biochemical activities of bone cells are considered relevant here. At any location within *Ω* _bm′_, these are determined as

| $\frac{\text{d}\text{C}_{\text{k}}^{\text{α}}}{\text{d}\text{t}}=\text{f}\left( \text{s}_{\text{k}\text{,}\text{α}}^{\text{h}} \right)$ | (5) |
| --- | --- |

where *f*(·) may involve an integration in order to account for signals in the neighbourhood and in history, $\text{C}_{\text{k}}^{\text{α}}$ is the local concentration of cell type *k* undergoing activity type *α* controlled by the biochemical signal $\text{s}_{\text{k,α}}^{\text{h}}$. Biochemical signals are taken to be dependent on the source (*h*) and to be known everywhere in *Ω*_bm′_. Here *k* takes values: *b* (osteoblast), *c* (osteoclast) and *y* (osteocyte); *α* takes values: *x* (apoptosis), *r* (replication) and *a* (activation); and *h* takes values: *A* (autocrine), *P* (paracrine) and *E* (endocrine).

At the instant of formation of a new bone matrix region, the phase transformation from bm′ to bm causes all cellular concentrations $\text{C}_{\text{k}}^{\text{α}}$ to go to zero and all biochemical signals $\text{s}_{\text{k,α}}^{\text{h}}$ to cease. At the same instant, the local material composition of this region becomes fixed in time and is determined by the biochemical activities occurring in the neighbourhood of that material location prior to the instant of phase-transformation. Here, for the sake of simplicity, only the determination of bone matrix density is explicitly stated

| $\text{ }\text{ρ}\text{ }\text{=}\text{ }\text{g}\left( \text{C}_{\text{k}}^{\text{α}} \right)$ | (6) |
| --- | --- |

where *g*(·) may include an integration similar to *f*(·). At the instant of an existing bone matrix region becoming fully resorbed, bone cell concentrations $\text{C}_{\text{k}}^{\text{α}}$ in the region are reset to zero and the spatial and temporal variation of biochemical signals $\text{s}_{\text{k,α}}^{\text{h}}$ are assumed to become known. Thus, in the absence of mechanical loading, the internal boundary d*Γ*_bm–bm′_ displaces by an amount *u_ρ_*. The mass of bone matrix material added or removed due to the movement of d*Γ*_bm–bm′_ over time d*t* is given by *ρ*${\dot{\text{u}}}_{\text{ρ}}$d*t*⋅*n*d*Γ*_bm–bm′_ where *ρ* is the local mass density of the bone matrix at the interface and *n* denotes the normal to the surface d*Γ*_bm–bm′_ pointing outward from *Ω*_bm_.

When both external surface tractions and displacements and biochemical activity are present, the above formulations can be reconciled by assuming a state of residual stress *R* to be present everywhere in *Ω* [27], and including a dependence of *H*^epd^ on *R*. To remain consistent with assumptions made previously, we take *R* = 0 everywhere in *Ω*_bm′_ (no ability to store mechanical energy) and *R* to remain constant with time everywhere in *Ω*_bm_ (biochemically inert), the non-zero value being determined by the biochemical activities occurring in the neighbourhood of that material location prior to the instant of phase-transformation. This determination can be described similar to equation (6) as

| $\text{ }\text{R}\text{ }\text{=}\text{ }\text{h}\left( \text{C}_{\text{k}}^{\text{α}} \right)$ | (7) |
| --- | --- |

The decoupling of mechanical and biochemical responses (by situating them in mutually distinct regions *Ω*_bm_ and *Ω*_bm′_ respectively) is an assumption made here only for simplification of expression. For instance, the function *f* may include a dependence on the mechanical response occurring in the part of the neighbourhood that falls in *Ω*_bm_. Indeed, this would achieve some degree of mechanoregulation of bone remodelling [28-30]. Similarly, a dependence of bone matrix material composition on biochemical activity in neighbouring non-bone matrix regions would allow bone matrix density to change even after phase transition. This would imply a change in constitutive law parameters (through equation (4)) and residual stresses for some time after phase-transition. Although potentially more realistic, such complex effects are ignored in the following analysis. Biochemical effects are considered on the remodelling of a bone that is otherwise in homeostasis.

Given the above abstraction of the closed system, femur fall strength *S* is defined as follows. Consider that at a given instant of interest, virtual surface tractions *τ* and displacements *a* are applied on the femur boundary *Γ*, such that the spatial distributions of *τ* and *a* represent a fall impact scenario. Consider the virtual force obtained by integrating *τ* over the part of the *Γ* that corresponds to the femur head. The smallest magnitude of this force that will cause the femur to fail mechanically is defined as its fall strength.

## Summary of the mathematical model

In the previous section, the bio-chemo-mechanical interactions at a single-scale of the bone region *Ω* were described. The model was formed on the basis of the following *idealisations*, simplifying assumptions that are necessary to reduce problem at hand to mathematically treatable terms:

1. The spatiotemporal domain of observation ranges between *l** = 10^–7^ m to *L** = 10^0^ m in length and from *t** = 10^–3^ s to *T** = 10^9^ s in time;
2. The region of bone comprises three material phases: an extracellular matrix phase, an extravascular phase and a vascular phase;
3. The boundary separating the bone matrix from the other two phases is a connected manifold and possesses features of typical length not smaller than *l**;
4. Load bearing occurs only in the bone matrix phase; the extravascular and vascular phases are always stress-free. In particular, hydraulic stiffening resulting from pore fluid flow within a connected extravascular phase, if it exists, is negligible relative to the stiffness of the bone matrix phase alone;
5. Biochemical activity leading to bone remodelling occurs only in the extravascular and vascular phases; the bone matrix phase is biochemically inert;
6. Under the action of externally-applied time-dependent loads and displacements, the bone matrix phase deforms but with a negligible rate of change of momentum;
7. The material forming the bone matrix determines its mechanical response; in particular, the undamaged elastic moduli tensor depends on bone matrix mass density;
8. The rate at which bone matrix material is apposed/resorbed depends on the local concentration of cells in the vascular and extra-vascular phases that are undergoing apoptosis, replication or activation;
9. The rate of change with time in cellular concentration is a function of cell-type- and process-specific signals originating from autocrine, paracrine and endocrine sources;
10. Conversely, the mass of the apposed/resorbed matrix material is unaffected by cell types, cellular processes and signalling sources not mentioned above;
11. The inter-phase boundary moves in space and time due to bone remodelling activity as material in the vascular and extra-vascular phases become bone matrix material (apposition) or vice-versa (resorption);
12. Mechanoregulation of biochemical activity is neglected; i.e. biochemical signalling and cellular activity is assumed to be independent of mechanical response.

With these assumptions, the full set of equations for the bio-chemo-mechanically active bone region is:

| Div *σ* = 0 | everywhere in *Ω*_bm_ | (H-i) |
| --- | --- | --- |
| $\dot{\text{σ}}$ = *H*^epd^(*σ*, *E*, *H*^(0)^, *K*, *d*, *R*)$\text{:}\dot{\text{E}}$ |  | (H-ii) |
| *H*^(0)^ = *H*^(0)^ (*ρ*) |  | (H-iii) |
| *F*∙*σ*∙*N* = *τ* | on the traction boundary *Γ*_bm,_*_τ_* | (H-iv) |
| *u* = *a* | on the displacement boundary *Γ*_bm,u_ | (H-v) |
| $\frac{\text{d}\text{C}_{\text{k}}^{\text{α}}}{\text{d}\text{t}}=\text{f}\left( \text{s}_{\text{k,α}}^{\text{h}} \right)$ | everywhere in *Ω*_bm′_ | (H-vi) |
| *F*∙*σ*∙*N* = 0 | on the internal boundary *Γ*_bm–bm′_ | (H-vii) |
| $\text{ }\text{ρ}\text{ }\text{=}\text{ }\text{g}\left( \text{C}_{\text{k}}^{\text{α}} \right)$ | on the internal boundary *Γ*_bm–bm′_ at the instant of phase transformation | (H-viii) |
| $\text{ }\text{R}\text{ }\text{=}\text{ }\text{h}\left( \text{C}_{\text{k}}^{\text{α}} \right)$ |  | (H-ix) |

The set of equations (H) in the unknowns *σ*, *u*, *H*^(0)^, *R*, $\text{C}_{\text{k}}^{\text{α}}$, *ρ* is closed. Given the distributions for *a* and *τ* and the distribution of $\text{s}_{\text{k,α}}^{\text{h}}$ throughout time and the initial distributions of the unknowns in space, the problem (H) set in the hypothetical scale can at least in principle be solved to obtain femur strength *S* at 10 years from the initial instant. However, both input and output variables vary over a very large spatiotemporal range. Thus, given the current limitations of experimental and computational instrumentation, in practice, it is not possible to inform or to solve the model.

## Empirical evidence of scale-dependent features

Bone cellular activity leading to bone remodelling can be measured in vivo using biochemical markers. There is evidence suggesting that biochemical marker measurements correlate with bone remodelling [31]. Histomorphometry of fluorochrome-labelled bone biopsies is the gold standard of experimental determination of bone remodelling and is best used to capture the dynamics of bone formation [32]. In adults, bone formation occurs in the last 4–6 months of an approximately 8-month long bone remodelling cycle [33]. Formation can be dynamically captured by labelling the bone surface at intervals of 8–14 days (8 days = 6.91 x 10^5^ s) [34, 35]. Changes in bone matrix morphology can also be quantified by comparing over durations of up to 3 months (= 90 days = 7.76 x 10^6^ s), which also correlate with changes in biochemical markers over similar periods [36]. Static and dynamic morphometric parameters are evaluated using a microscope eyepiece with typical resolution 0.1 μm [6]. For adult human bone, the largest changes of interest typically span 0.2 mm in length (e.g. change in average trabecular thickness over a 3-month period [36]). These distances and time spans provide the grain and extent for a real scale labelled S1.

State-of-the-art microCT can image bone regions with distances up to 80 mm at a nominal isotropic resolution of 10 μm [14]. MicroCT cannot be used to image human femurs in vivo. Yet, ex vivo studies on cadavers have captured changes in bone tissue morphology with time showing the evidence of bone remodelling [37], and bone tissue morphology measured by microCT is known to be a key determinant of whole bone strength [38]. The maximum duration over which bone tissue morphological changes are of interest to measure is the 10-year period (= 3650 days = 3 x 10^8^ s) used for clinical assessment of fracture risk [39]. Effect of ageing was measured using cadaveric specimens by categorizing specimens by age determined to within one year (= 365 days = 3 x 10^7^ s) [37]. These distances and time spans provide the grain and extent of scale S2.

The adult human femur is on average 44 cm long [40, 41]. Measurement of femur strength is possible only ex vivo. Well-established protocols exist that can be used to assess ex vivo bone strength under relevant loading conditions, such as one that will replicate a spontaneous fracture in an elderly subject [42]. The most accurate models that predict ex vivo measurements of bone strength use clinical quantitative CT data obtained at a resolution of 0.625 mm [15, 43]. Impact forces applied on the femur surface (such as during a fall to the side) and strains within the femur possess characteristic temporal variations in the range of 1–1000 ms [11, 12]. These distances and time spans provide the grain and extent of scale S3.

The three scales “S1”, “S2” and “S3” are used to populate the hypothetical scale described up to the previous section. For simplicity, distances and time spans used to define grain and extent have been rounded down and rounded up, respectively, to the nearest power of 10.

Thus, for the adult human femur:

1. Scale S1, *l**_1_ = 10^–7^ m, *t**_1_ = 10^5^ s, *L**_1_ = 10^–3^ m, *T**_1_ = 10^7^ s
2. Scale S2, *l**_2_ = 10^–5^ m, *t**_2_ = 10^7^ s, *L**_2_ = 10^–1^ m, *T**_2_ = 10^9^ s
3. Scale S3, *l**_3_ = 10^–4^ m, *t**_3_ = 10^–3^ s, *L**_3_ = 10^0^ m, *T**_3_ = 10^0^ s

The corresponding scale separation map is shown in Fig 5A of the main text.

## Modelling assumptions revisited

Following the scale separation defined as above, the single-scale model idealisations listed in section S2.3 can be reduced to a smaller, but equivalent, set of idealizations at each scale. These assumptions are given in the following.

Scale S1

1. The domain of observation ranges from 10^–7^ m to 10^–3^ m in length and from 10^5^ s to 10^7^ s in time;
2. The region of bone comprises three material phases: an extracellular matrix phase (bone matrix), an extravascular phase and a vascular phase;
3. The volume occupied by the bone matrix phases is connected; the volume occupied by the extravascular and vascular phases is connected; the boundary separating the bone matrix from the other two phases is a connected manifold and possesses features of typical length not smaller than 10^–7^ m;
4. Load bearing occurs only in the bone matrix phase; the extravascular and vascular phases are always stress-free. In particular, hydraulic stiffening resulting from pore fluid flow within a connected extravascular phase, if it exists, is negligible relative to the stiffness of the bone matrix phase alone;
5. Biochemical activity leading to bone remodelling occurs only in the extravascular and vascular phases; the bone matrix phase is biochemically inert;
6. Externally applied loads and displacements averaged over S1 time scales are negligible; hence phase transformation induced residual stresses in the bone matrix phase are also negligible;
7. The material forming the bone matrix determines its mechanical response; in particular, the undamaged elastic moduli tensor depends on bone matrix mass density;
8. The rate at which bone matrix material is apposed/resorbed depends on the local concentration of cells in the vascular and extra-vascular phases that are undergoing apoptosis, replication or activation;
9. The rate of change with time in cellular concentration is a function of cell-type- and process-specific signals originating from autocrine, paracrine and endocrine sources;
10. Conversely, the mass of the apposed/resorbed matrix material is unaffected by cell types, cellular processes and signalling sources not mentioned above;
11. The inter-phase boundary moves in space and time due to bone remodelling activity as material in the vascular and extra-vascular phases become bone matrix material (apposition) or vice-versa (resorption);
12. The movement of the boundary and the apposition/resorption of bone matrix occurs over a characteristic time *t*_1_ (*t**_1_ = 10^5^ s < *t*_1_ < *T**_1_ = 10^7^ s);
13. Mechanoregulation of biochemical activity is neglected; i.e. biochemical signalling and cellular activity is assumed to be independent of mechanical response of bone matrix phase.

Scale S2

1. The domain of observation ranges from 10^–5^ m to 10^–1^ m in length and from 10^7^ s to 10^9^ s in time;
2. The region of bone comprises two distinct material phases: one phase that is purely vascular material and another (mixed phase) that comprises extracellular matrix, extravascular and vascular material but the boundaries between which cannot be distinguished at this scale;
3. The volume occupied by the S2 scale mixed phase *Ω*_[2]m_ is connected; the boundary separating the purely vascular phase from the mixed phase *Γ*_[2]m–[2]v_ possesses features of typical length not smaller than 10^–5^ m;
4. The purely vascular material is stress-free and is active only biochemically, the mixed phase is responsive both mechanically and biochemically;
5. The spatial heterogeneity of the constitutive properties of the mixed phase arises from two sources: the distribution of the three phases comprising it and the constitutive properties of the bone matrix phase;
6. The constitutive properties of the mixed phase display significant anisotropy; this arises due to the topological orientation of the bone matrix phase averaged over a S2 scale volume;
7. Within the mixed phase, constitutive properties change with time due to the movement of the inter-phase boundary *Γ*_bm–bm′_;
8. The boundary between the purely vascular and the mixed phases moves when observed over a characteristic time *t*_2_ (*t**_2_ = 10^7^ s < *t*_2_ < *T**_2_ = 10^9^ s);
9. Externally applied loads and displacements averaged over S2 scale times are negligible; due to this reason, and due to negligible residual stresses in the bone matrix phase at the scale S1, the residual stresses in the S2 scale mixed phase due to phase transformation are also negligible.

Scale S3

1. The domain of observation ranges from 10^–4^ m to 10^0^ m in length and from 10^–3^ s to 10^0^ s in time;
2. The region of bone comprises two distinct material phases: one phase that is purely vascular material and another (mixed phase) that comprises the two S2 scale material phases, but the boundaries between which cannot be distinguished at this scale;
3. The volume occupied by the S3 scale mixed phase *Ω*_[3]m_ is connected; the boundary separating the purely vascular phase from the mixed phase *Γ*_[3]m–[3]v_ possesses features of typical length not smaller than 10^–4^ m;
4. The purely vascular material is stress-free and is active only biochemically, the mixed phase is responsive both mechanically and biochemically;
5. The spatial heterogeneity of the constitutive properties of the S3 scale mixed phase arises from two sources: the distribution of the two phases comprising it and the constitutive properties of the S2 scale mixed phase;
6. The constitutive properties of the mixed phase are isotropic; the topological orientation of the S2 scale mixed phase averaged over S3 scale volumes possesses negligible anisotropy;
7. The constitute behaviour of the mixed phase can be described as linear elastic with failure occurring without plastic yield at small strains;
8. Residual stresses in the S3 scale mixed phase are negligible due to negligible residual stresses in the S2 scale mixed phase;
9. Averaged over S3 scale volumes and observed at characteristic S3 scale times, changes in bone cellular concentrations in the vascular phase can be neglected; thus, the constitutive properties of the S3 scale mixed phase are constant over S3 scale times.

## Revised component hypomodels

The revised set of assumptions above lead to the revision of the mathematical models at each scale (component hypomodels), along with redefinitions for each of the variables of the hypothetical scale model as follows.

Scale S1

At the scale S1, the bone matrix phase volume is denoted by *Ω*_bm_, the volume of remaining two phases is jointly denoted by *Ω*_bm′_ and the boundary separating the two volumes is denoted by *Γ*_bm–bm′_. The external boundary of the volume *Ω*_bm_ comprises a part on which external tractions are applied (*Γ*_bm,_*_τ_*) and a part on which external displacement are applied (*Γ*_bm,_*_u_*). At any location within *Ω*_bm_, *ρ*^[1]^, *H*^epd[1]^, *H*^(0)[1]^, *d*^[1]^ and *K*^[1]^ refer respectively to the mass density, elastoplastic damage moduli tensor, undamaged elastic moduli tensor, damage evolution variable and yield stress. At any location within *Ω*_bm′_, $\text{C}_{\text{k}}^{\text{α}}$ and $\text{s}_{\text{k,α}}^{\text{h}}$ refer to bone cell concentrations and biochemical signals. The following are assumed to be known at the initial instant: geometries of volumes *Ω*_bm_ and *Ω*_bm′_ and of surfaces *Γ*_bm–bm′_, *Γ*_bm,_*_τ_* and *Γ*_bm,_*_u_*, the distribution of *ρ*^[1]^ within *Ω*_bm_ and the distribution of $\text{C}_{\text{k}}^{\text{α}}$ within *Ω*_bm′_. The distributions of *K*^[1]^ and *d*^[1]^ within *Ω*_bm_, and the distributions of $\text{s}_{\text{k,α}}^{\text{h}}$ within *Ω*_bm′_ are assumed to be known at all times. The S1 scale model is:

| *H*^epd[1]^ = *H*^epd[1]^ (*σ*^[1]^, *E*^[1]^, *H*^(0)[1]^, *K*^[1]^, *d*^[1]^) | everywhere in *Ω*_bm_ | (S1-i) |
| --- | --- | --- |
| *H*^(0)[1]^ = *H*^(0)[1]^ (*ρ*^[1]^) |  | (S1-ii) |
| $\frac{\text{d}\text{C}_{\text{k}}^{\text{α}}}{\text{d}\text{t}}=\text{f}\left( \text{s}_{\text{k,α}}^{\text{h}} \right)$ | everywhere in *Ω*_bm′_ | (S1-iii) |
| $\text{ρ}^{\left[ \text{1} \right]}\text{ }\text{=}\text{ }\text{g}\left( \text{C}_{\text{k}}^{\text{α}} \right)$ | on the internal boundary *Γ*_bm–bm′_ at the instant of phase transformation | (S1-iv) |

The solution to this model yields the spatial and temporal distributions of *H*^(0)[1]^ and *ρ*^[1]^ within *Ω*_bm_ and the spatial and temporal distributions $\text{C}_{\text{k}}^{\text{α}}$ within *Ω*_bm′_. As a result, it is possible to evaluate *H*^epd[1]^ at any location and time in dependence of the dummy variables *σ*^[1]^ and *E*^[1]^ only, which represent the second PK stress and the GL strain at any location in the bone matrix phase. As stated before, stresses and strains in the bone matrix phase are negligible *when observed* at scale S1; these quantities should not be confused with the dummy variables. The reason for considering these dummy variables will become clear later.

Scale S2

At the scale S2, the mixed phase volume is denoted by *Ω*_[2]m_, the purely vascular phase volume by *Ω*_[2]v_, and the boundary separating the two phases by *Γ*_[2]m–[2]v_. The external boundary of the volume *Ω*_[2]m_ comprises a part on which external tractions are applied (*Γ*_[2]m,_*_τ_*) and a part on which external displacement are applied (*Γ*_[2]m,_*_u_*). At any location within *Ω*_[2]m_, *ρ*^[2]^, *H*^epd[2]^, *H*^(0)[2]^, *K*^[2]^ and *d*^[2]^ denote the average mass density of the bone matrix phase, elastoplastic damage moduli tensor, undamaged elastic moduli tensor, yield stress and damage evolution variable respectively. The following are assumed to be known at all instants: geometries of volumes *Ω*_tm_ and *Ω*_tv_ and of surfaces *Γ*_[2]m–[2]v_, *Γ*_[2]m,_*_τ_* and *Γ*_[2]m,_*_u_*, and distributions of *ρ*^[2]^, *K*^[2]^ and *d*^[2]^ within *Ω*_[2]m_. The S2 scale model is:

| *H*^epd[2]^ = *H*^epd[2]^ (*σ*^[2]^, *E*^[2]^, *H*^(0)[2]^, *K*^[2]^, *d*^[2]^) | everywhere in *Ω*_[2]m_ | (S2-i) |
| --- | --- | --- |
| *H*^(0)[2]^ = *H*^(0)[2]^ (*ρ*^[2]^) |  | (S2-ii) |

The solution to this model yields the spatial and temporal distributions of *H*^(0)[2]^ within *Ω*_bm_, and *H*^epd[2]^ at any location and time in dependence of the dummy variables *σ*^[2]^ and *E*^[2]^ only, which represent the second PK stress and the GL strain at any location in *Ω*_[2]m_. As stated before, stresses and strains in *Ω*_[2]m_ are negligible *when observed* at scale S2; these quantities should not be confused with the dummy variables. The reason for considering these dummy variables will become clear later.

Scale S3

At the S3 scale, the mixed phase volume is denoted by *Ω*_[3]m_, the purely vascular phase volume by *Ω*_[3]v_, the boundary separating the two phases by *Γ*_[3]m–[3]v_. The external boundary of the volume *Ω*_[3]m_ comprises a part on which external tractions are applied (*Γ*_[3]m,_*_τ_*) and a part on which external displacement are applied (*Γ*_[3]m,_*_u_*). The local outward normal at any boundary of the mixed phase volume is denoted by *N*^[3]^. At any location within *Ω*_[3]m_, *ρ*^[3]^, *σ*^[3]^, *ε*^[3]^ = ½ (Grad *u*^[3]^ + (Grad *u*^[3]^)^T^), *H*^(0)[3]^ and *u*^[3]^ denote, respectively, the average mass density of bone matrix phase, the Cauchy stress tensor, the infinitesimal strain tensor, the undamaged elastic modulus tensor and the displacement. Externally-imposed surface tractions and displacements are denoted by *τ*^[3]^ and *a*^[3]^ respectively. A failure surface *Φ* is defined with respect to the failure strain *γ*^[3]^. The following are assumed to be known as constant with time: geometries of volumes *Ω*_[3]m_ and *Ω*_[3]v_ and of surfaces *Γ*_[3]m–[3]v_, *Γ*_[3]m,_*_τ_* and *Γ*_[3]m,_*_u_* and the distributions of *ρ*^[3]^ and *γ*^[3]^ within *Ω*_[3]m_. The spatiotemporal distributions of *τ*^[3]^ and *a*^[3]^ on the boundaries *Γ*_om,_*_τ_* and *Γ*_om,_*_u_* respectively are assumed to be known. The organ scale model is:

| Div *σ*^[3]^ = 0 | everywhere in *Ω*_[3]m_ | (S3-i) |
| --- | --- | --- |
| *σ*^[3]^ = *H*^(0)[3]^:*ε*^[3]^ for *Φ*(*ε*^[3]^, *γ*^[3]^) < 0 |  | (S3-ii) |
| *H*^(0)[3]^ = *H*^(0)[3]^ (*ρ*^[3]^) |  | (S3-iii) |
| *σ*^[3]^*∙N*^[3]^ *= τ*^[3]^ | on the traction boundary *Γ*_[3]m,_*_τ_* | (S3-iv) |
| *u*^[3]^ = *a*^[3]^ | on the displacement boundary *Γ*_[3]m,u_ | (S3-v) |
| *σ*^[3]^*∙N*^[3]^ *=* 0 | on the internal boundary *Γ*_[3]m–[3]v_ | (S3-vi) |

The solution to this model yields the spatiotemporal distributions of *H*^(0)[3]^, *σ*^[3]^, *u*^[3]^ and *ε*^[3]^ within *Ω*_bm_.

The multiscale problem requires all models (S1), (S2) and (S3) to be solved together. The following are assumed to be known at the initial instant: geometries of volumes *Ω*_bm_, *Ω*_lac_ and *Ω*_vas_, the distribution of *ρ*^[1]^ within *Ω*_bm_ and the distribution of $\text{C}_{\text{k}}^{\text{α}}$ within *Ω*_bm′_ = *Ω*_lac_ U *Ω*_vas_. The following are assumed to be known at all times: the distributions of *K*^[1]^ and *d*^[1]^ within *Ω*_bm_, the distributions of $\text{s}_{\text{k,α}}^{\text{h}}$ within *Ω*_bm′_ and the partitioning of the bounding surface *Γ*_[3]m_ into *Γ*_[3]m,_*_τ_* and *Γ*_[3]m,_*_u_*. The distributions of *τ*^[3]^ and *a*^[3]^ on *Γ*_[3]m,_*_τ_* and *Γ*_[3]m,_*_u_* respectively are assumed to be constant with time and known. Note that the volume definitions automatically determine the external and inter-phase boundaries. In order to close the multiscale problem, additional equations are needed to determine the geometries of volumes *Ω*_[2]m_, *Ω*_[2]v_, *Ω*_[3]m_ and *Ω*_[3]v_ and distributions of *ρ*^[2]^, *K*^[2]^ and *d*^[2]^ within *Ω*_[2]m_, and the distributions of *ρ*^[3]^ and *γ*^[3]^ within *Ω*_[3]m_. This requires relation models to be developed.

## Relation models

By definition, any location in *Ω*_[2]v_ is mapped to a region that lies fully within *Ω*_vas_. The mapping from *Ω*_vas_ to *Ω*_[2]v_ satisfies the additional constraint that the total volume occupied by *Ω*_[2]v_ is the largest possible given its spatial resolution, although the set of sub-volumes comprising *Ω*_[2]v_ need not be connected. Algorithmically, this mapping is similar to geometry-smoothing operations in mesh generation or shrink-wrap operations in image processing. Once *Ω*_[2]v_ is known, we get *Ω*_[2]m_ = *Ω* \ *Ω*_[2]v_ and from this definition *Ω*_[2]m_ can be mapped to regions comprising *Ω*_bm_ and *Ω*_bm′_. Finally, *Γ*_[2]m–[2]v_ is defined as the boundary common to *Ω*_[2]m_ and *Ω*_[2]v_. It is expected that locations on *Γ*_[2]m–[2]v_ will lie within an *l**_2_-neighbourhood of ∂*Ω*_vas_. Having related the geometries between scales, *ρ*^[3]^ can be computed from *ρ*^[2]^ using a volume-averaging operation. Determination of constitutive properties *K*^[2]^ and *d*^[2]^ at any location *x*^[2]^ in within *Ω*_[2]m_ is typically performed by reducing the variability of S1-scale constitutive properties within *x*^[2]^ to that of a repeating pattern (a representative volume element, or RVE, approach). Irrespective of whether this variability is homogenized using an analytical or numerical approach, *a priori* knowledge is required of the constitutive relationship at scale S1, relating an arbitrary applied stress (or strain) to the resulting strain (or stress). The dummy variables *σ*^[1]^ and *E*^[1]^ introduced in the S1-scale component hypomodel take these arbitrary stress/strain values. Mathematically, the above relation models are expressed as:

| *Γ*_[2]m–[2]v_ = *Γ*_[2]m–[2]v_ (*Ω*_bm_, *Ω*_bm′_) | in the *l**_2_-neighbourhood of ∂*Ω*_vas_ | (R12-i) |
| --- | --- | --- |
| *ρ*^[2]^ = *ρ*^[2]^ (*ρ*^[1]^, *Ω*_bm_, *Ω*_bm′_) | everywhere in *Ω*_[2]m_ | (R12-ii) |
| *K*^[2]^ *=* *K*^[2]^ (*H*^(0)[1]^, *K*^[1]^, *d*^[1]^, *Ω*_bm_, *Ω*_bm′_) |  | (R12-iii) |
| *d*^[2]^ *= d*^[2]^ (*H*^(0)[1]^, *K*^[1]^, *d*^[1]^, *Ω*_bm_, *Ω*_bm′_) |  | (R12-iv) |

Using an identical set of arguments, the relation models from scale S2 to scale S3 are as follows:

| *Γ*_[3]m–[3]v_ = *Γ*_[2]m–[2]v_ (*Ω*_[2]m_, *Ω*_[2]v_) | in the *l**_3_-neighbourhood of ∂*Ω*_[2]v_ | (R23-i) |
| --- | --- | --- |
| *ρ*^[3]^ = *ρ*^[3]^ (*ρ*^[2]^, *Ω*_[2]m_, *Ω*_[2]v_) | everywhere in *Ω*_[3]m_ | (R23-ii) |
| *γ*^[3]^ *= γ*^[3]^ (*H*^(0)[2]^, *K*^[2]^, *d*^[2]^, *Ω*_[2]m_, *Ω*_[2]v_) |  | (R23-iii) |

Together, the component hypomodels (S1), (S2) and (S3) and relation models (R12) and (R23) constitute a closed set of multiscale model equations describing the mechanobiology of an adult femur.

## Orchestration

The orchestration for the multiscale model elaborated above is shown in Fig 5B of the main text. The model (S1) involves an integration with time in order to update the distribution of the bone matrix phase in response to bone remodelling. The S1 scale properties are homogenised in space and time using model (R12) to obtain material and mechanical property distributions at scale S2, which involves executing model (S2). Model (R23) is used to homogenise these properties to obtain material and mechanical property distributions at scale S1. Executing model (S1) using this data along with the data at scale S1 on applied displacement constraints and load distribution leads to the determination of stresses and strains at scale S1 in dependence of any applied distribution of surface tractions *τ* and displacements *a*. Thus, femur fall strength can be determined by computing the crossing of the yield surface *Φ*. As the time-marching of the solution is controlled by the S1 scale model, the predicted bone strength at the end of the 10-year period is also obtained at a temporal resolution of *t**_1_. Note that the final result does not depend on the intermediate solution variables *H*^epd[1]^ and *H*^epd[2]^. Hence, one may choose to not solve equations (S1-i) and (S2-i) without affecting the result. We chose to retain these in the above model such that the framework can be extended to incorporate mechanoregulation of bone remodelling, although this would require additional modifications to the model orchestration.

# Femur strength after 8 weeks in a living mouse

We now consider the problem of predicting the strength of a mouse femur at the end of a period of 8 weeks (= 56 days = 4.84 x 10^6^ s), which is the typical duration of preclinical studies investigating effects of drugs or other interventions [44]. Note that the typical length of femurs of adult mice (~15 mm) is about 100x smaller than the typical length of adult human femurs [45]. Consider a mouse femur B occupying a connected region *Ω* in **R**^3^ with a maximum size *L** = 10^–2^ m and comprising the three material phases previously elaborated. The vascular phase is found only in the bone marrow due to the absence of a Haversian system in mouse bone [2]. The boundary *Γ*_bm–bm′_ separating the bone matrix and non-bone matrix phases is assumed to possess features of characteristic length larger than *l** = 10^–7^ m. For adult mouse bones, this includes trabecular spacing [~ 579 μm, see 46] and lacunar porosities diameter [15±15 μm, see 47] but excludes canaliculi [130–390 nm in diameter in the tibia, see 48, 49]. It is assumed that it is sufficient to measure the variation in traction magnitudes applied on the bone surface to within *t** = 0.04 s in order to capture femur strength [1]. Thus, the domain of observation ranges from 10^–7^ m to 10^–2^ in length, and from 10^–2^ s to 10^7^ s in time. With these differences accounted for, the abstraction of the closed system and the mathematical formulation of the hypothetical scale model remain the same as before. However, the empirical evidence of scale-dependence of features is substantially different.

Firstly, changes in mouse femur microstructure are measured typically by histomorphometric analysis. Bone surfaces are labelled sequentially at 5-day intervals [44], and the histomorphometric measures correlate well with serum concentrations of biochemical markers of bone turnover. The biomarkers show changes when measured at intervals as small as 2 days (= 1.73 x 10^5^ s) and the average trabecular thickness (42 μm) provides an upper limit for the largest feature that is of interest to measuring bone formation [44]. Thus, a scale (labelled S1′) can be defined with *l**_1_ = 10^–7^ m, *t**_1_ = 10^5^ s, *L**_1_ = 10^–4^ m, *T**_1_ = 10^7^ s. Secondly, femur strength is commonly evaluated using a three-point bending test. The total experimental duration based on typical applied loading rates is about 5 s. van Lenthe et al. [50] used microCT images of bone taken at a resolution of 20 μm to predict the stiffness of mouse femurs in three-point bending. Oliviero et al. [51] used a micro-finite-element (FE) modelling approach similar to that of van Lenthe et al. [50] to predict the full strain field in mouse tibia under axial compression. The microFE model of Oliviero et al. [51] was based on microCT images taken at a resolution of 10 μm and the strain predictions were validated using digital volume correlation. The 10 μm microCT resolution has been shown to be achievable in vivo for mouse bones [52]. As the strain determines the point of failure of bone, it is assumed that in order to predict mouse femur strength in vivo, a microFE model based on 10 μm resolution microCT would be necessary. Thus, a second scale (labelled S2′) is defined with *l**_2_ = 10^–5^ m, *t**_2_ = 10^–2^ s, *L**_2_ = 10^–2^ m, *T**_2_ = 10^1^ s. For simplicity, distances and time spans used to define grain and extent have been rounded down and rounded up, respectively, to the nearest power of 10. Fig 6A in the main text shows the corresponding scale separation map. This scale separation leads to the following two component hypomodels at scales S1′ and S2′:

| *H*^epd[1]^ = *H*^epd[1]^ (*σ*^[1]^, *E*^[1]^, *H*^(0)[1]^, *K*^[1]^, *d*^[1]^) | everywhere in *Ω*_bm_ | (S1′-i) |
| --- | --- | --- |
| *H*^(0)[1]^ = *H*^(0)[1]^ (*ρ*^[1]^) |  | (S1′-ii) |
| $\frac{\text{d}\text{C}_{\text{k}}^{\text{α}}}{\text{dt}}=\text{f}\left( \text{s}_{\text{k,α}}^{\text{h}} \right)$ | everywhere in *Ω*_bm′_ | (S1′-iii) |
| *ρ*^[1]^ = *g*$\left( \text{C}_{\text{k}}^{\text{α}} \right)$ | on the internal boundary *Γ*_bm–bm′_ at the instant of phase transformation | (S1′-iv) |

and

| Div *σ*^[2]^ = 0 | everywhere in *Ω*_[2]m_ | (S2′-i) |
| --- | --- | --- |
| *σ*^[2]^ = *H*^[2]^:*ε*^[2]^ for *Φ*(*ε*^[2]^, *γ*^[2]^) < 0 |  | (S2′-ii) |
| *H*^[2]^ = *H*^[2]^ (*ρ*^[2]^) |  | (S2′-iii) |
| *σ*^[2]^*∙N*^[2]^ *= Τ*^[2]^(*t*) | on the traction boundary *Γ*_[2]m,_*_τ_* | (S2′-iv) |
| *u*^[2]^ = *a*^[2]^(*t*) | on the displacement boundary *Γ*_[2]m,u_ | (S2′-v) |
| *σ*^[2]^*∙N*^[2]^ *=* 0 | on the internal boundary *Γ*_[2]m–[2]v_ | (S2′-vi) |

These are supplemented by relation hypomodels

| *Γ*_[2]m–[2]v_ = *Γ*_[2]m–[2]v_ (*Ω*_bm_, *Ω*_bm′_) | in the *l**_2_-neighbourhood of ∂*Ω*_vas_ | (R12′-i) |
| --- | --- | --- |
| *ρ*^[2]^ = *ρ*^[2]^ (*ρ*^[1]^, *Ω*_bm_, *Ω*_bm′_) | everywhere in *Ω*_[2]m_ | (R12′-ii) |
| *γ*^[2]^ *= γ*^[2]^ (*H*^(0)[1]^, *K*^[1]^, *d*^[1]^, *Ω*_bm_, *Ω*_bm′_) |  | (R12′-iii) |

The model orchestration is shown in Fig 6B of the main text.

# References

1. Jepsen KJ, Silva MJ, Vashishth D, Guo XE, van der Meulen MC. Establishing biomechanical mechanisms in mouse models: practical guidelines for systematically evaluating phenotypic changes in the diaphyses of long bones. J Bone Miner Res. 2015;30(6):951-66.

2. Fritsch A, Hellmich C. ‘Universal’ microstructural patterns in cortical and trabecular, extracellular and extravascular bone materials: Micromechanics-based prediction of anisotropic elasticity. J Theor Biol. 2007;244:597-620. doi: 10.1016/j.jtbi.2006.09.013.

3. Vaughan TJ, Verbruggen SW, McNamara LM. Are all osteocytes equal? Multiscale modelling of cortical bone to characterise the mechanical stimulation of osteocytes. Int J Numer Meth Bio. 2013;29:1361-72. doi: 10.1002/cnm.2578.

4. Cardoso L, Fritton SP, Gailani G, Benalla M, Cowin SC. Advances in assessment of bone porosity, permeability and interstitial fluid flow. J Biomech. 2013;46(2):253-65. doi: 10.1016/j.jbiomech.2012.10.025.

5. Ren L, Yang P, Wang Z, Zhang J, Ding C, Shang P. Biomechanical and biophysical environment of bone from the macroscopic to the pericellular and molecular level. J Mech Behav Biomed Mater. 2015;50:104-22. doi: 10.1016/j.jmbbm.2015.04.021.

6. Revell PA. Histomorphometry of bone. J Clin Pathol. 1983;36(12):1323-31. doi: 10.1136/jcp.36.12.1323.

7. Bernhard A, Milovanovic P, Zimmermann EA, Hahn M, Djonic D, Krause M, et al. Micro-morphological properties of osteons reveal changes in cortical bone stability during aging, osteoporosis, and bisphosphonate treatment in women. Osteoporos Int. 2013;24(10):2671-80. doi: 10.1007/s00198-013-2374-x.

8. Currey JD. Some Effects of Ageing in Human Haversian Systems. J Anat. 1964;98(1):69-75.

9. Carter Y, Thomas CD, Clement JG, Peele AG, Hannah K, Cooper DM. Variation in osteocyte lacunar morphology and density in the human femur--a synchrotron radiation micro-CT study. Bone. 2013;52(1):126-32. doi: 10.1016/j.bone.2012.09.010.

10. Marotti G. The original contributions of the scanning electron microscope to the knowledge of bone structure. In: Bonucci E, Motta PM, editors. Ultrastructure of Skeletal Tissues: Bone and Cartilage in Health and Disease. Electron Microscopy in Biology and Medicine. 7. Boston, MA: Springer US; 1990. p. 19-39.

11. Burr DB, Milgrom C, Fyhrie D, Forwood M, Nyska M, Finestone A, et al. In vivo measurement of human tibial strains during vigorous activity. Bone. 1996;18(5):405-10. doi: 10.1016/8756-3282(96)00028-2.

12. Robinovitch SN, McMahon TA, Hayes WC. Force attenuation in trochanteric soft tissues during impact from a fall. J Orthop Res. 1995;13(6):956-62. doi: 10.1002/jor.1100130621.

13. Langer M, Pacureanu A, Suhonen H, Grimal Q, Cloetens P, Peyrin F. X-ray phase nanotomography resolves the 3D human bone ultrastructure. PLoS One. 2012;7(8):e35691. doi: 10.1371/journal.pone.0035691.

14. Zimmermann EA, Schaible E, Gludovatz B, Schmidt FN, Riedel C, Krause M, et al. Intrinsic mechanical behavior of femoral cortical bone in young, osteoporotic and bisphosphonate-treated individuals in low- and high energy fracture conditions. Sci Rep. 2016;6:21072. doi: 10.1038/srep21072.

15. Qasim M, Farinella G, Zhang J, Li X, Yang L, Eastell R, et al. Patient-specific finite element estimated femur strength as a predictor of the risk of hip fracture: the effect of methodological determinants. Osteoporos Int. 2016;27(9):2815–22 doi: 10.1007/s00198-016-3597-4.

16. Anderson DD, Brown TD, Radin EL. Stress wave effects in a finite element analysis of an impulsively loaded articular joint. Proc Inst Mech Eng H. 1991;205(1):27-34. doi: 10.1243/PIME_PROC_1991_205_258_02.

17. Malvern LE. Introduction to the mechanics of a continuous medium. Englewood Cliffs, NJ: Prentice-Hall; 2007.

18. Marsden JE, Hughes TJR. Mathematical foundations of elasticity. New York: Dover; 1994.

19. Perić D, Owen DRJ, Honnor ME. A model for finite strain elasto-plasticity based on logarithmic strains: Computational issues. Comput Methods Appl Mech Eng. 1992;94(1):35-61. doi: 10.1016/0045-7825(92)90156-e.

20. Rho J-Y, Tsui TY, Pharr GM. Elastic properties of human cortical and trabecular lamellar bone measured by nanoindentation. Biomaterials. 1997;18(20):1325-30. doi: 10.1016/s0142-9612(97)00073-2.

21. Hasegawa K, Turner CH, Burr DB. Contribution of collagen and mineral to the elastic anisotropy of bone. Calcif Tissue Int. 1994;55(5):381-6. doi: 10.1007/bf00299319.

22. Korsa R, Lukes J, Sepitka J, Mares T. Elastic properties of human osteon and osteonal lamella computed by a bidirectional micromechanical model and validated by nanoindentation. J Biomech Eng. 2015;137(8):081002. doi: 10.1115/1.4030407.

23. Nalla RK, Kinney JH, Ritchie RO. Mechanistic fracture criteria for the failure of human cortical bone. Nat Mater. 2003;2(3):164-8. doi: 10.1038/nmat832.

24. Carretta R, Stussi E, Muller R, Lorenzetti S. Within subject heterogeneity in tissue-level post-yield mechanical and material properties in human trabecular bone. J Mech Behav Biomed Mater. 2013;24:64-73. doi: 10.1016/j.jmbbm.2013.04.014.

25. Rho JY, Zioupos P, Currey JD, Pharr GM. Variations in the individual thick lamellar properties within osteons by nanoindentation. Bone. 1999;25(3):295-300. doi: 10.1016/s8756-3282(99)00163-5.

26. Ju JW. Energy‐based coupled elastoplastic damage models at finite strains. J Eng Mech. 1989;115(11):2507-25. doi: 10.1061/(asce)0733-9399(1989)115:11(2507).

27. Ciarletta P, Destrade M, Gower AL. On residual stresses and homeostasis: an elastic theory of functional adaptation in living matter. Sci Rep. 2016;6:24390. doi: 10.1038/srep24390.

28. Chamay A, Tschantz P. Mechanical influences in bone remodeling. Experimental research on Wolff's law. J Biomech. 1972;5(2):173-80. doi: 10.1016/0021-9290(72)90053-x.

29. Iolascon G, Resmini G, Tarantino U. Mechanobiology of bone. Aging Clin Exp Res. 2013;25 Suppl 1:S3-7. doi: 10.1007/s40520-013-0101-2.

30. Rubin CT, Lanyon LE. Regulation of bone formation by applied dynamic loads. J Bone Joint Surg Am. 1984;66(3):397-402.

31. Chavassieux P, Portero-Muzy N, Roux JP, Garnero P, Chapurlat R. Are biochemical markers of bone turnover representative of bone histomorphometry in 370 postmenopausal women? J Clin Endocrinol Metab. 2015;100(12):4662-8. doi: 10.1210/jc.2015-2957.

32. Compston JE. Histomorphometric interpretation of bone biopsies for the evaluation of osteoporosis treatment. Bonekey Rep. 2012;1:47. doi: 10.1038/bonekey.2012.47.

33. Clarke B. Normal bone anatomy and physiology. Clin J Am Soc Nephro. 2008;3:S131-S9. doi: 10.2215/CJN.04151206

34. Hauge EM, Mosekilde L, Melsen F, Frydenberg M. How many patients are needed? Variation and design considerations in bone histomorphometry. Bone. 2001;28(5):556-62. doi: 10.1016/s8756-3282(01)00424-0.

35. Kimmel DB, Recker RR, Gallagher JC, Vaswani AS, Aloia JF. A comparison of iliac bone histomorphometric data in post-menopausal osteoporotic and normal subjects. Bone Miner. 1990;11(2):217-35. doi: 10.1016/0169-6009(90)90061-j.

36. Dobnig H, Sipos A, Jiang Y, Fahrleitner-Pammer A, Ste-Marie LG, Gallagher JC, et al. Early changes in biochemical markers of bone formation correlate with improvements in bone structure during teriparatide therapy. J Clin Endocrinol Metab. 2005;90(7):3970-7. doi: 10.1210/jc.2003-1703.

37. Turunen MJ, Prantner V, Jurvelin JS, Kroger H, Isaksson H. Composition and microarchitecture of human trabecular bone change with age and differ between anatomical locations. Bone. 2013;54(1):118-25. doi: 10.1016/j.bone.2013.01.045.

38. Verhulp E, van Rietbergen B, Huiskes R. Load distribution in the healthy and osteoporotic human proximal femur during a fall to the side. Bone. 2008;42(1):30-5. doi: 10.1016/j.bone.2007.08.039.

39. Kanis JA, Oden A, Johansson H, Borgstrom F, Strom O, McCloskey E. FRAX and its applications to clinical practice. Bone. 2009;44(5):734-43. doi: 10.1016/j.bone.2009.01.373.

40. Cristofolini L, Viceconti M, Cappello A, Toni A. Mechanical validation of whole bone composite femur models. J Biomech. 1996;29(4):525-35.

41. Gardner MP, Chong AC, Pollock AG, Wooley PH. Mechanical evaluation of large-size fourth-generation composite femur and tibia models. Ann Biomed Eng. 2010;38(3):613-20. doi: 10.1007/s10439-009-9887-7.

42. Cristofolini L, Juszczyk M, Martelli S, Taddei F, Viceconti M. In vitro replication of spontaneous fractures of the proximal human femur. J Biomech. 2007;40(13):2837-45. doi: 10.1016/j.jbiomech.2007.03.015.

43. Altai Z, Qasim M, Li X, Viceconti M. The effect of boundary and loading conditions on patient classification using finite element predicted risk of fracture. Clin Biomech (Bristol, Avon). 2019;68:137-43. doi: 10.1016/j.clinbiomech.2019.06.004.

44. Pierroz DD, Bonnet N, Baldock PA, Ominsky MS, Stolina M, Kostenuik PJ, et al. Are osteoclasts needed for the bone anabolic response to parathyroid hormone? A study of intermittent parathyroid hormone with denosumab or alendronate in knock-in mice expressing humanized RANKL. J Biol Chem. 2010;285(36):28164-73. doi: 10.1074/jbc.M110.101964.

45. Jamsa T, Jalovaara P, Peng Z, Vaananen H, Tuukkanen J. Comparison of three-point bending test and peripheral quantitative computed tomography analysis in the evaluation of the strength of mouse femur and tibia. Bone. 1998;23(2):155-61. doi: 10.1016/s8756-3282(98)00076-3.

46. Weinstein RS, Jilka RL, Parfitt AM, Manolagas SC. The effects of androgen deficiency on murine bone remodeling and bone mineral density are mediated via cells of the osteoblastic lineage. Endocrinology. 1997;138(9):4013-21. doi: 10.1210/endo.138.9.5359.

47. Carriero A, Doube M, Vogt M, Busse B, Zustin J, Levchuk A, et al. Altered lacunar and vascular porosity in osteogenesis imperfecta mouse bone as revealed by synchrotron tomography contributes to bone fragility. Bone. 2014;61:116-24. doi: 10.1016/j.bone.2013.12.020.

48. You LD, Weinbaum S, Cowin SC, Schaffler MB. Ultrastructure of the osteocyte process and its pericellular matrix. Anat Rec A Discov Mol Cell Evol Biol. 2004;278(2):505-13. doi: 10.1002/ar.a.20050.

49. Muller LO, Toro EF. A global multiscale mathematical model for the human circulation with emphasis on the venous system. Int J Numer Method Biomed Eng. 2014;30(7):681-725. doi: 10.1002/cnm.2622.

50. van Lenthe GH, Voide R, Boyd SK, Muller R. Tissue modulus calculated from beam theory is biased by bone size and geometry: implications for the use of three-point bending tests to determine bone tissue modulus. Bone. 2008;43(4):717-23. doi: 10.1016/j.bone.2008.06.008.

51. Oliviero S, Giorgi M, Dall'Ara E. Validation of finite element models of the mouse tibia using digital volume correlation. J Mech Behav Biomed Mater. 2018;86:172-84. doi: 10.1016/j.jmbbm.2018.06.022.

52. Oliviero S, Lu Y, Viceconti M, Dall'Ara E. Effect of integration time on the morphometric, densitometric and mechanical properties of the mouse tibia. J Biomech. 2017;65:203-11. doi: 10.1016/j.jbiomech.2017.10.026.
