# Supplementary material for: A systematic approach to the scale separation problem in the development of multiscale models
Source: PLoS One. 2021 May 18;16(5):e0251297. doi: 10.1371/journal.pone.0251297 (PMC8130972; doi:10.1371/journal.pone.0251297)
Supplement: Attachment — Submitted filename: Review of PONER1.pdf [file pone.0251297.s005.pdf]

Review of PONE-D-20-14710R1

The reviewer appreciates that the authors have provided a quite comprehensive response to the questions raised in the first round of reviews, complemented by some additional remarks in the revised manuscript. While still not described in minute detail, the authors seem to roughly perform the following computing steps for the human femur analysis, by combining fitting functions with linear elastic FE analysis:

(i) Calibration of CT-Hounsfield values to volumetric bone mineral density (depicted in Figures 1 and 4), (ii) probably relating the latter to apparent mass density[1], in order to then (iii) use REF 41 - Morgan et al's fitting relationship between apparent mass density and loading modulus; (iv) use of corresponding modulus values in linear elastic Finite Element analysis, in order to compute principal strains; (v) averaging the strain values over pre-set regions of interest, and (vi) inserting the latter into a strain-based failure criterion[2]. In their response, the authors stress that this approach was validated in Taddei et al, 2007, *Med Eng Phys 29, 973-979, 2007*; however, the latter reference uses Keller's[3] 1994 density-to-modulus relationship (rather than the relationship of Morgan et al) and obviously *ex vivo* experiments.

In this context, the reviewer, being asked for a rigorous review, re-iterates on several, so far hardly addressed critical scientific issues, which – in one way or another – were already referred to in the first round of reviews:

- Mass-density-to-modulus fitting functions reflecting corresponding experimental data on human femoral bone show very large variations, and differ from each other by several hundred percent; as reviewed in *Clin Biomech 23, 135–146, 2008*, see in particular Figure 4(a).
- The aforementioned moduli were determined in ***loading* mode**. This entails a critical issue concerning elasticity in the sense of continuum mechanics and thermodynamics, see e.g. well-known landmark contributions such as *Coleman and Noll, The Foundations of Mechanics and Thermodynamics, pp. 145-156, 1974; Coussy, Poromechanics, Wiley, 2004; Rajagopal, Z angew Math Phys 58, 309–317, 2007,* and references therein: *elasticity* is associated with the conversion of internal energy into efficient mechanical work. Mechanical work is only gained during the unloading phase, which is indeed characterized by *higher* (truely elastic) moduli, see e.g. *J Mech Beh Biomed Mat 52, 51-62, 2015.* Hence, the authors' comparison, in response to Comment R2.2., of fitted *loading* moduli with micromechanics-predicted elasticity tensor components (validated by the fastest ultrasonics waves, which are always associated with elasticity, see e.g. *Carcione, Wave Fields in Real Media, Oxford, 2001*) lacks scientific rigour.
- The authors unarguably use the **scientific language and mathematical formalisms of continuum mechanics** (e.g. Cauchy stress tensor and elastic stiffness tensor on page 10), and at the same time, they write, in response to Comment R2.4, "the approach presented in this manuscript should in no way be confused with the AM&CM (standing for applied mathematics and continuum mechanics). Already from a purely logical viewpoint, this does not make sense.
- More importantly, the stress tensor, as originally introduced by Cauchy in the 1820s, is connected to forces acting centrally on facets of a small *solid* element (as can be found in virtually any textbook on continuum mechanics, see e.g. *Salençon, Handbook of Continuum Mechanics, Springer, 2001; Fung et al, Classical and Computational Mechanics, 2017*), and it was not before 1941, that a satisfying extension of the stress tensor concept towards porous materials was proposed, namely by M.A.Biot, first in the context of soils (quote from *Journal of Applied Physics 12, 155ff. 1941*): *"Consider a small cubic element of the consolidating soil,*

[1] The authors write that REF 32 Altai et al would give information on CT data processing, however, the reviewer could not even find the abbreviation „CT" in this paper.
[2] It would *really* helpful to state this more explicitly in the manuscript, in the context of Eq.(5).
[3] J Biomech 1994;27(9):1159–68.

its sides being parallel with the coordinate axes. This element is taken to be <u>large enough compared to the size of the pores</u> so that it may be treated as homogeneous, and at the same time <u>small enough, compared to the scale of the macroscopic phenomena</u> in which we are interested, so that it may be considered as infinitesimal in the mathematical treatment." Similar definitions were given in the framework of continuum micromechanics in the 1960s. Hence, the reviewer is not aware of any well-accepted historical or conceptual grounds based on which the stress tensor may be applied to porous materials (such as bone), other than elements similar to the aforementioned "small cubic element", i.e. RVEs in the currently dominating terminology. Thanks to the seminal work of Cowin and colleagues, see e.g. *J Biomech 32, 217-238, 1999*, this reasoning has become a major theoretical fundament in bone biomechanics as well.

- Beyond that, and practically speaking, the use of Cauchy's stress tensor has major consequences for the **scale of instrumentation l*** (a central concept put forward by the authors), both in ultrasonic tests and mechanical tests, as discussed next.
- According to page 6 of the manuscript, "one can ignore variations separated by distances l* or smaller." Similarly, on page 3 of the supplementary material, one can read "for any two points that are separated by a distance smaller than l*, variables of interest taken at these points (e.g. location, stress, traction, displacement and strain) cannot be distinguished from each other". In other words, at the scale l*, the quantities *do not vary with space* - they are *homogeneous*. Accordingly, **scale l*** in ultrasonic tests is not the sample size (as assumed by the authors when deploring "the discrepancy of scales compared by Hellmich et al [3]" in their response to Comment R2.2), but is **governed by the fluctuation length / wavelength**, see (again) e.g. *J Eng Mech 128, 808-816, 2002 or J Theor Biol 244, 597–620, 2007* for the requirement l << L, which needs to hold for any continuum formulation (including that of the authors). Only for lengths sufficiently smaller than the wave length, the scale l* is reached. Accordingly, **MHz-frequency** tests typically relate to material **scales l*** (RVE-sizes) **much smaller than the ultrasonic sample size**. Conclusively, one sees that the **instrumentation scale l*** should definitely not be arbitrarily chosen, but **needs to comply with the employed physical/mechanical premises** (e.g. equations of motions, plane waves, Cauchy stress etc.).
- The situation with mechanical test is similar. If homogeneous traction forces are prescribed on the boundaries of a sample (as in carefully designed uniaxial testing), the sample size is indeed l*. In case of bending tests with stress gradients, as done by Morgan et al, l* needs to be sufficiently small with respect to structural loading scale (sigma / grad sigma), so that the l* (and correspondingly tested RVE) is again smaller than the sample size.
- In contrast to the authors statements on line 80 of the revised manuscript, all continuum micromechanics models discussed by the authors do not imply time and space periodicity. The authors need to distinguish between far-field based homogenization approaches (without any type of periodicity required) and asymptotic homogenization approaches.
- Somehow at odds with the authors' statements on the open literature, CT-informed coupled X-ray-physics-micromechanics models were applied to the human femur problem and validated against experiments on whole femurs, see e.g. *Int J Multiscale Comp Eng 6, 483-498, 2008; J Biomech Eng. 133, 061001, 2011*; and corresponding *elastoplastic* approaches for vertebra bones have been shown to agree with *in vivo* fracture cases observed in athletes, see *Int J Numer Meth Biomed Engng 32, e02760, 2016.*

The reviewer has given these statements for the sake of a transparent scientific discussion. The authors may find these remarks useful when addressing a larger scientific community, going beyond the confines of traditional bone biomechanics. Needless to say, any decisions on the suitability of the manuscript for PLoS ONE are entirely in the hands of the handling editor.
