# Supplementary material for: A systematic approach to the scale separation problem in the development of multiscale models
Source: PLoS One. 2021 May 18;16(5):e0251297. doi: 10.1371/journal.pone.0251297 (PMC8130972; doi:10.1371/journal.pone.0251297)
Supplement: Attachment — Submitted filename: Response to Reviewers.docx [file pone.0251297.s006.docx]

# 1. Responses to Reviewer #2 comments

Comment R2.1

As the authors' proposition for the scale separation problem is in conflict with major premises of continuum mechanics, a more detailed review was necessary, and is attached as a separate file [*Author’s Note: This is appended below*].

The reviewer appreciates that the authors have provided a quite comprehensive response to the questions raised in the first round of reviews, complemented by some additional remarks in the revised manuscript. While still not described in minute detail, the authors seem to roughly perform the following computing steps for the human femur analysis, by combining fitting functions with linear elastic FE analysis:

(i) Calibration of CT-Hounsfield values to volumetric bone mineral density (depicted in Figures 1 and 4);
(ii) probably relating the latter to apparent mass density, in order to then;

[*Reviewer #2’s Note A: The authors write that REF 32 Altai et al would give information on CT data processing, however, the reviewer could not even find the abbreviation “CT” in this paper*]

(iii) use REF 41-Morgan et al’s fitting relationship between apparent mass density and loading modulus;
(iv) use of corresponding modulus values in linear elastic Finite Element analysis, in order to compute principal strains;
(v) averaging the strain values over pre-set regions of interest, and
(vi) inserting the latter into a strain-based failure criterion.

[*Reviewer #2’s Note B: It would really helpful to state this more explicitly in the manuscript, in the context of Eq.(5).*]

In their response, the authors stress that this approach was validated in Taddei et al, 2007, Med Eng Phys 29, 973-979, 2007; however, the latter reference uses Keller’s 1994 [*Reviewer #2’s Note C*: *J Biomech 1994; 27(9):1159-68*] density-to-modulus relationship (rather than the relationship of Morgan et al) and obviously ex vivo experiments. In this context, the reviewer, being asked for a rigorous review, re-iterates on several, so far hardly addressed critical scientific issues, which – in one way or another – were already referred to in the first round of reviews.

Response

The reviewer has correctly outlined the FE analysis approach to predict bone strength.

However, we would like to point out that in §1 Introduction of Altai et al (2019), the last sentence of the first paragraph reads: “*The most investigated alternative method is the quantitative-computed-tomography (****QCT****)-based subject-specific finite element modelling (FE) (Viceconti et al., 2018)*”. There are three other mentions of the abbreviation CT in the main text of the article (excluding references).

More importantly, the first paragraph of §2.2 Finite element modelling in Altai et al. (2019) explains how the FE model is obtained from CT scans. As this is a well-established methodology, hence Altai et al (2019) refer to past papers when explaining the method. This FE modelling method is not the novelty of the present manuscript. Hence, we refer the reader to Altai et al (2019) (Ref 31 in the present manuscript) for the details. We believe that repeating these details could potentially distract a reader from the main message of our multiscale modelling approach. Following the reviewer’s suggestion, on lines 246–247 we have clarified the use of the strain-based failure criterion in Eq. (1) (we believe “Eq. (5)” in the comment is a misprint as that equation describes the change in bone strength over the 10-year period).

In response to Comment R2.2 in the last revision, it was stated: “*In the in vivo setting, multiple studies [13-16] have shown that the regression model of Morgan et al. [7] coupled with an FE modelling approach based on Taddei et al. [9] predicts bone strength values that can classify fracture status very accurately.*” To imply from this statement that Taddei et al used the regression result of Morgan et al is a misunderstanding.

Comment R2.2

1. Mass-density-to-modulus fitting functions reflecting corresponding experimental data on human femoral bone show very large variations, and differ from each other by several hundred percent; as reviewed in Clin Biomech 23, 135–146, 2008, see in particular Figure 4(a).

Response

Helgason et al (Clin Biomech 23, 135–146, 2008) point out that for a given anatomical site, the measurement of mechanical response is affected by experimental conditions e.g. specimen size and shape and end support. As the reviewer points out, this is the uncertainty in the measurement data, and should not be confused with prediction accuracy of a given regression model.

Helgason et al note that a cylindrical sample with 2:1 aspect ratio analysed using the end-cap technique leads to the highest accuracy and precision. Once all these experimental conditions are met – as in the studies of Morgan et al (2003) – one can assess the error in using the regression model to predict the mechanical response. As mentioned in response to Comment R2.2 (previous revision), this error is ~ 443 MPa for Morgan et al (2003) fitting function, and smaller than that of the Hellmich et al (2003) model. The bone density to elasticity relationship used in the present study is that of Morgan et al (2003); this is already specified on line 242 of the manuscript and papers cited therein (Refs 40 and 41 in particular).

As Comment R2.2 concerns only our response in the last revision, and not any statement in the manuscript itself, no corresponding changes to the manuscript have been made.

Comment R2.3

2. The aforementioned moduli were determined in loading mode. This entails a critical issue concerning elasticity in the sense of continuum mechanics and thermodynamics, see e.g. well-known landmark contributions such as Coleman and Noll, The Foundations of Mechanics and Thermodynamics, pp. 145-156, 1974; Coussy, Poromechanics, Wiley, 2004; Rajagopal, Z Angew Math Phys 58, 309–317, 2007, and references therein: elasticity is associated with the conversion of internal energy into efficient mechanical work. Mechanical work is only gained during the unloading phase, which is indeed characterized by higher (truely elastic) moduli, see e.g. J Mech Beh Biomed Mat 52, 51-62, 2015. Hence, the authors’ comparison, in response to Comment R2.2, of fitted loading moduli with micromechanics-predicted elasticity tensor components (validated by the fastest ultrasonics waves, which are always associated with elasticity, see e.g. Carcione, Wave Fields in Real Media, Oxford, 2001) lacks scientific rigour.

Response

Our response to Comment R2.2 (previous revision) responded partly to the suggestion in Comment R2.1 (previous revision) that multiscale problems in bone biomechanics have already been “largely solved”. Thus, we identified the problem of predicting bone strength over a 10-year period. The articles suggested by the reviewer in Comment R2.2 (previous revision) contained several models for bone tissue mechanics. At the point of writing the response, we were unaware of a FE modelling pipeline that employs micromechanics-based elasticity tensors (please see Comment R2.8 below and our response to the same). Hence, to further the reviewer’s argument, we inserted the micromechanics-based elasticity tensor components into our finite element modelling pipeline which employs fitted moduli measured in loading mode. Our response did not seek to compare fitted loading moduli with micromechanics-predicted elasticity tensor components, but comparison of errors in FE predictions based on these. Our analysis suggested that “it is doubtful that if the model of Hellmich et al. [3] was used to predict femur strength, the resulting accuracy would be higher than that of the S1 scale model of §2.3.” On this basis, we differed from the reviewer’s opinion that there is no need for a new multiscale modelling approach as multiscale problems in bone biomechanics have been “largely solved”.

The above conclusion remains unchanged upon reanalysing the situation in view of the papers kindly pointed out by the reviewer. Details are given in response to Comment R2.8.

As Comment R2.3 concerns only our response in the last revision, and not any statement in the manuscript itself, no corresponding changes to the manuscript have been made.

Comment R2.4

3. The authors unarguably use the scientific language and mathematical formalisms of continuum mechanics (e.g. Cauchy stress tensor and elastic stiffness tensor on page 10), and at the same time, they write, in response to Comment R2.4, “*the approach presented in this manuscript should in no way be confused with the AM&CM*” (standing for applied mathematics and continuum mechanics). Already from a purely logical viewpoint, this does not make sense.

Response

Applied Mathematics and Continuum Mechanics (AM&CM) is a label. No label can fully explain what the scientific precepts of a method are (or are not). Formal definitions of the collection of multiscale modelling approaches to which AM&CM refers can be found in papers such as Fish (2006) and Geers et al (2010) (Refs 9 and 10 in the manuscript). In addition, throughout the manuscript we define and describe our multiscale modelling approach and discuss in detail how it differs from the AM&CM approach. As such, we clearly state in line 110–112 that we expect the present multiscale modelling approach to be applied largely in situations where continuum mechanics is the only appropriate physical idealisation. It is therefore our opinion that in to understand what the scientific precepts of the current approach are, a reader will not need to draw any inferences from the “AM&CM” label itself (or even any parts or permutations of it).

As Comment R2.4 concerns only our response in the last revision, and not any statement in the manuscript itself, no corresponding changes to the manuscript have been made.

Comment R2.5

4. More importantly, the stress tensor, as originally introduced by Cauchy in the 1820s, is connected to forces acting centrally on facets of a small solid element (as can be found in virtually any textbook on continuum mechanics, see e.g. Salençon, Handbook of Continuum Mechanics, Springer, 2001; Fung et al, Classical and Computational Mechanics, 2017), and it was not before 1941, that a satisfying extension of the stress tensor concept towards porous materials was proposed, namely by M.A.Biot, first in the context of soils (quote from Journal of Applied Physics 12, 155ff. 1941): “Consider a small cubic element of the consolidating soil, its sides being parallel with the coordinate axes. This element is taken to be large enough compared to the size of the pores so that it may be treated as homogeneous, and at the same time small enough, compared to the scale of the macroscopic phenomena in which we are interested, so that it may be considered as infinitesimal in the mathematical treatment.” Similar definitions were given in the framework of continuum micromechanics in the 1960s. Hence, the reviewer is not aware of any well-accepted historical or conceptual grounds based on which the stress tensor may be applied to porous materials (such as bone), other than elements similar to the aforementioned “*small cubic element*”, i.e. RVEs in the currently dominating terminology. Thanks to the seminal work of Cowin and colleagues, see e.g. J Biomech 32, 217-238, 1999, this reasoning has become a major theoretical fundament in bone biomechanics as well.

Response

We agree fully with this comment. Indeed, we are operating under the same mathematical and mechanical precepts. The divergence is in the application of these concepts, as detailed in the response to the following comment. The manuscript remains unchanged in response to this particular comment.

Comment R2.6

5. Beyond that, and practically speaking, the use of Cauchy’s stress tensor has major consequences for the scale of instrumentation l* (a central concept put forward by the authors), both in ultrasonic tests and mechanical tests, as discussed next.
6. According to page 6 of the manuscript, “*one can ignore variations separated by distances l* or smaller.”* Similarly, on page 3 of the supplementary material, one can read “*for any two points that are separated by a distance smaller than l*, variables of interest taken at these points (e.g. location, stress, traction, displacement and strain) cannot be distinguished from each other.*” In other words, at the scale l*, the quantities do not vary with space - they are homogeneous. Accordingly, scale l* in ultrasonic tests is not the sample size (as assumed by the authors when deploring “*the discrepancy of scales compared by Hellmich et al [3]*” in their response to Comment R2.2), but is governed by the fluctuation length / wavelength, see (again) e.g. J Eng Mech 128, 808-816, 2002 or J Theor Biol 244, 597–620, 2007 for the requirement l << L, which needs to hold for any continuum formulation (including that of the authors). Only for lengths sufficiently smaller than the wavelength, the scale l* is reached. Accordingly, MHz-frequency tests typically relate to material scales l* (RVE-sizes) much smaller than the ultrasonic sample size. Conclusively, one sees that the instrumentation scale l* should definitely not be arbitrarily chosen, but needs to comply with the employed physical/mechanical premises (e.g. equations of motions, plane waves, Cauchy stress etc.).
7. The situation with mechanical test is similar. If homogeneous traction forces are prescribed on the boundaries of a sample (as in carefully designed uniaxial testing), the sample size is indeed l*. In case of bending tests with stress gradients, as done by Morgan et al, l* needs to be sufficiently small with respect to structural loading scale (sigma / grad sigma), so that the l* (and correspondingly tested RVE) is again smaller than the sample size.

Response

Point #5 can be alternatively restated as: the use of Cauchy’s stress tensor has major consequences on the accuracy of the model when the scale is fixed by the choice of instrumentation. This is our preference, as it allows to choose the experiment arbitrarily, to quantify the error in prediction (which are bound to result from neglecting the requirements mentioned in #6 and #7), and to propagate this error through the model – all methodologically rigorous and scientifically valid steps. The error is quantified by comparing model prediction with observation at each component scale. Verification, validation and uncertainty quantification of the present approach are fully detailed in lines 635–712 of the manuscript.

Our preference as mentioned above is motivated as follows. Even for the most carefully constructed experiment, conditions of “homogeneity” are attained in the limit. Deviations from this condition always exist, even though these are finitely small. We argue that constraining the instrumentation (while carefully constructing the experiment) can limit the scenarios in which the resulting multiscale model can be applied.

As Comment R2.6 concerns only our response in the last revision, and not any statement in the manuscript itself, no corresponding changes to the manuscript have been made.

Comment R2.7

8. In contrast to the authors statements on line 80 of the revised manuscript, all continuum micromechanics models discussed by the authors do not imply time and space periodicity. The authors need to distinguish between far-field based homogenization approaches (without any type of periodicity required) and asymptotic homogenization approaches.

Response

We have amended the statements on lines 72, 79, 85, 113 and 116 in the revision to include macrohomogeneity as the basis for mean field homogenization approaches.

Comment R2.8

9. Somehow at odds with the authors’ statements on the open literature, CT-informed coupled X-ray-physics-micromechanics models were applied to the human femur problem and validated against experiments on whole femurs, see e.g. Int J Multiscale Comp Eng 6, 483-498, 2008; J Biomech Eng. 133, 061001, 2011; and corresponding elastoplastic approaches for vertebra bones have been shown to agree with in vivo fracture cases observed in athletes, see Int J Numer Meth Biomed Engng 32, e02760, 2016.

The reviewer has given these statements for the sake of a transparent scientific discussion. The authors may find these remarks useful when addressing a larger scientific community, going beyond the confines of traditional bone biomechanics. Needless to say, any decisions on the suitability of the manuscript for PLoS ONE are entirely in the hands of the handling editor.

Response

In the previous revision the reviewer kindly referred us to some papers by Hellmich et al. as examples of micromechanics (MM)-based multiscale modelling approaches. The main argument was that these works have to a great extent solved the multiscale challenge in bone mechanics. In our response to that Comment R2.2 (previous version) we raised a doubt against this argument – and detailed whether an MM-model (such as detailed in these papers) would lead to a more accurate prediction of femur strength than the empirical model used to derive constitutive properties in the present approach. The doubt was based on the observation that errors in predicting true MM-derived elastic moduli were in excess of errors in predicting the stiffness measured in loading mode using empirical relationships (such as those of Morgan et al). We postulated that this larger uncertainty in quantifying elasticity could potentially balance out improvements due to the consideration of orthotropy in material response as informed by a micromechanics approach.

At that time of writing the previous response, we were indeed unaware of the abovementioned FE analyses of human femur based on MM-derived moduli. We thank the reviewer for pointing out these additional papers by Trabelsi and co-workers. Yet, these papers only confirm our previous conclusion. Although based on a limited study (in all, 5 cadaveric femurs), the more recent article, Trabelsi et al (J Biomech Eng. 133, 061001, 2011) conclude that “*The differences between the isotropic model* [i.e. empirical model] *and the orthotropic MM model* [with regard to displacements and strains] *are in the range of the experimental errors so unequivocal conclusions cannot be drawn regarding which model is better*”. The explanatory remarks in square brackets […] are ours. Note that in the clinical problem considered in the illustrations in the present study, the final objective is to predict femur strength, and the above cited studies conducted by Trabelsi and co-workers do not predict femur strength. As such, MM-based models cannot be taken to be the “final word” on multiscale modelling of femur strength, and there remains an unmet need to develop new multiscale modelling methods.

We hope that a response to the remark regarding models of vertebra is not needed as their performance has little bearing on the femur strength problem.

As Comment R2.8 concerns only our response in the last revision, and not any statement in the manuscript itself, no corresponding changes to the manuscript have been made.

# 2. Responses to Reviewer #4 comments

Comment R4.1

This paper addresses an important topic of scale separation in multiscale modeling of materials. However, the reviewer has general comments on how to improve this paper. The literature review is rather limited and overlooking some important topics and works.

1. The scale separation in modeling is certainly an important condition, and it is tacitly assumed in most studies. The idea of “grain” and “extent” is novel and linking scales to imaging equipment scale is practical and interesting. However, the other important concept closely related but untouched in the paper is the concept of a representative volume element. The term is not mentioned in the paper except for a reference by Drugan and Willis, which is not representative (their results only hold for periodic microstructures). Another important aspect is material randomness, which again is fully overlooked. A comprehensive reference on these three topics (scale separation, representative volume element, and randomness) is, for example, a recent book (A. Malyarenko and M. Ostoja-Starzewski, Tensor-Valued Random Fields for Continuum Physics, Cambridge University Press, 2019), which summarizes past works in this area. The reviewer does not recommend incorporating these concepts in the model, but these topics merit at least mentions.

Response

We thank the reviewer for their comment. We already discussed the term scale separation at length in the manuscript – indeed it is in the title of the manuscript and the focus of the introduction section. In the revised version, we have included a brief discussion of the concepts of representative volume element and material randomness on lines 78–81.

Comment R4.2

2. Secondly, the authors are citing in their revised paper about ten papers by the Hellmich group, which were not present in the original version of the article. They do not mention other similar works employing micromechanics approaches. The reviewer recommends that the references on analytical micromechanics formulations are more balanced, not favoring a specific research group.

The model is relatively crude, but the incorporation of both spatial and temporal scales in this simple model is a substantial contribution. Also, the example of a femur modeled over ten years is of high clinical interest. The ideas presented in this paper should stimulate more realistic models of bone and may stimulate research in other areas.
In summary, the reviewer recommends this paper for publication after the points raised above are addressed.

Response

In the revised version, we cite only one paper by the Hellmich group (Ref 15) and have included another paper from a different research group (Ref 16) on line 85. Although there are several research groups working on analytical micromechanics formulations, we chose these works as they also discuss bone mechanics, in order to set the context for the illustrative examples.
